# Development and Quality Evaluation of Fiber‐Enriched Pasta Incorporating Green Banana Flour and Whole Wheat Flour: Physicochemical, Antioxidant, and Sensory Properties

**DOI:** 10.1002/fsn3.72273

**Published:** 2026-08-27

**Authors:** Shariful Islam, Nusrat Sadia Himu, Md. Faridul Islam, Amena Kibria, Mohammad Nazrul Islam Bhuiyan, Md. Jaynal Abedin, Md. Nuruddin Miah, Md. Abdus Satter Miah

**Affiliations:** ^1^ Institute of Food Science and Technology (IFST) Bangladesh Council of Scientific and Industrial Research (BCSIR) Dhaka Bangladesh; ^2^ Department of Biochemistry and Molecular Biology Sher e Bangla Agricultural University Dhaka Bangladesh; ^3^ Department of Food Technology and Nutritional Science Mawlana Bhashani Science and Technology University Tangail Bangladesh; ^4^ BCSIR Chattogram Laboratories, BCSIR Chattogram Bangladesh; ^5^ Biomedical and Toxicological Research Institute (BTRI) Bangladesh Council of Scientific and Industrial Research (BCSIR) Dhaka Bangladesh

**Keywords:** antioxidant activity, cooking quality, dietary fiber enrichment, functional pasta, physicochemical properties, sensory evaluation

## Abstract

This study evaluated the effects of incorporating green banana and whole wheat flour at varying substitution levels on the nutritional, functional, antioxidant, cooking, and sensory properties of formulated novel pasta. Six formulations were developed and compared against a commercial control. Proximate analysis revealed significant enhancements in enriched pasta, with protein increasing from 13.85 g/100 g in the control to 16.12 g/100 g in WCL_20%_, and dietary fiber more than doubling from 3.9 g/100 g to 10.1–11.2 g/100 g in the developed pasta. Mineral contents also improved substantially, with calcium rising from 172.6 mg/100 g to 292.9 mg/100 g and iron from 2.7 mg/100 g to 17.1 mg/100 g. Antioxidant evaluation confirmed marked increases, with total phenolic content peaking at 27.8 μg gallic acid equivalent/g dry weight in BCL_20%_ and DPPH radical scavenging improving from 13.4% in the control to 95.7% in WCL_20%_. Cooking characteristics showed slightly higher water uptake and cooking loss in enriched formulations. Texture analysis revealed increased hardness from 11.3 × 10^−1^ N in control to 13.6 × 10^−1^ N in BCL_20%_ and stickiness, while sensory evaluation indicated that 10%–15% enrichment levels achieved the highest acceptability scores. Water activity values (0.49–0.55) across all samples were below the microbial safety threshold (< 0.60), suggesting stable shelf life. Therefore, green banana and whole wheat flours significantly improved pasta's nutritional and antioxidant properties, with optimal substitution at 10%–15% to maximize health benefits while maintaining consumer preference.

## Graphical abstract

This study developed fiber‐enriched pasta by incorporating green banana flour and whole wheat flour and evaluated its nutritional, physicochemical, antioxidant, and sensory properties. Enrichment increased protein, dietary fiber, calcium, and iron contents, while DPPH radical‐scavenging activity reached 95.7%. The incorporation of these flours caused slight increases in cooking loss and water uptake. Among the formulations, 10% substitution provided the best balance between nutritional improvement and sensory acceptability. All formulations showed water activity below 0.60, indicating good microbial stability. The findings demonstrate the potential of green banana and whole wheat flours for developing nutritionally enhanced pasta.

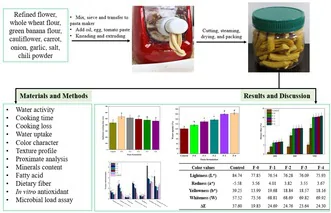

## 1 Introduction

Pasta is a globally consumed staple food, and cherished for its convenience, sensory appeal, and versatility. Conventionally produced from refined wheat flour, traditional pasta is predominantly composed of carbohydrates and offers limited dietary fiber and bioactive components, making it a nutritionally poor choice when consumed frequently (Kaur et al. 2023). With the growing burden of non‐communicable diseases (NCDs) linked to poor diet quality such as obesity, type‐2 diabetes, and cardiovascular disorders, there is a mounting demand for functional foods that deliver health benefits without compromising taste or consumer satisfaction (WHO 2024).

Dietary fiber, a key nutritional component, plays an important role in reducing the risk of chronic diseases and supporting gastrointestinal health. Epidemiological and clinical studies have demonstrated that fiber intake is inversely associated with the incidence of type‐2 diabetes, colorectal cancer, and cardiovascular disease (Anderson et al. 2009; Nweze et al. 2021). Despite its proven benefits, fiber consumption remains below recommended levels in many populations, especially in urban areas undergoing dietary transitions, such as Dhaka, Bangladesh. Integrating fiber‐rich ingredients into staple foods such as pasta can be a viable strategy to bridge this gap while catering to modern dietary habits.

To enhance the nutritional and functional quality of pasta, researchers have explored various plant‐based fiber sources, including fruit and vegetable residues, bran fractions, and tuber flours (Elleuch et al. 2011; Kyro and Tjonneland 2016; Plakantonaki et al. 2023). These sources not only contribute to dietary fiber but are also rich in polyphenols, flavonoids, and vitamin compounds known for their antioxidant activity and health‐promoting effects. Functional pasta formulations enriched with ingredients such as apple pomace (Gumul et al. 2023), barley β‐glucan (Palafox‐Carlos et al. 2011), and resistant starch (Munir et al. 2024) have demonstrated improvements in antioxidant potential and glycemic response. However, these nutritional improvements often come with trade‐offs in texture, color, and cooking performance, highlighting the need for optimized formulation strategies.

In Bangladesh, the popularity of pasta has surged due to changing urban lifestyles, increasing exposure to Western dietary patterns, and a preference for ready‐to‐cook products. Local pasta market remains underdeveloped, with limited domestic production and high dependency on imported or branded products that are often unaffordable for the general population. At the same time, Bangladesh possesses a diverse array of underutilized plant resources such as cauliflower, green bananas, and whole wheat that are rich in fiber and antioxidants and available at relatively low cost. Leveraging these local ingredients for functional food development offers both economic and nutritional benefits.

Cauliflower ( *Brassica oleracea var. botrytis* ), a cruciferous vegetable, contributes glucosinolates and dietary fiber known for their anti‐inflammatory and anticancer properties (Nartea et al. 2023). Whole wheat flour retains the bran and germ portions of the grain, providing a robust source of insoluble fiber, B vitamins, and minerals (Slavin 2005). Onion and garlic powders are rich in sulfur‐containing compounds, flavonoids, and phenolics that contribute to antioxidant activity, flavor enhancement, and potential antimicrobial effects in pasta formulations. Their inclusion not only improves the sensory profile but also supports the development of functional foods with extended shelf life and added health benefits (Sunanta et al. 2023). Meanwhile, green banana flour is high in resistant starch and has shown potential in improving digestive health and lowering postprandial glycemic response (Karaman et al. 2025). When used in pasta formulations, these ingredients can simultaneously improve nutritional value and enhance the product's functional properties.

Nevertheless, challenges remain regarding the impact of fiber enrichment on the physicochemical and cooking properties of pasta. Increased fiber levels may lead to changes in dough structure, water absorption, and gelatinization behavior, thereby influencing parameters such as cooking time, cooking loss, texture, and color (Rodriguez‐Huezo et al. 2022). Similarly, the antioxidant properties of enriched pasta depend on the retention of phenolics and other bioactive compounds during processing, which may vary based on ingredient type and level of incorporation (Betrouche et al. 2022). In the context of a growing processed food market in Bangladesh projected to reach USD 2.87 billion in pasta sales by 2025 with a 64.8% annual growth rate (Statista 2024), developing low‐cost, nutritionally superior pasta using local raw materials is both timely and impactful. Such products can support national health goals, foster innovation in food processing, and provide a model for integrating underutilized crops into functional food systems.

Although several studies have investigated pasta enrichment using individual ingredients such as green banana flour or whole wheat flour, limited information is available regarding their combined application and its effects on physicochemical, cooking, nutritional, antioxidant, and sensory properties. Given these considerations, this study was undertaken to examine the effects of incorporating selected dietary fiber and antioxidant sources at varying levels into pasta formulations. The investigation emphasized changes in nutritional composition, color and texture, antioxidant activity (including total phenolics, flavonoids, overall antioxidant capacity, and free radical scavenging potential), cooking performance (optimum cooking time, water uptake, and cooking loss), and sensory quality.

## 2 Materials and Methods

### 2.1 Experimental Raw Materials

Green banana, cauliflower, onion, garlic, tomato were purchased from kawran bazar, Dhaka and transported to Institute of food science and technology (IFST), BCSIR, Dhaka. Edible parts of vegetables except for tomato were cut into thin slices and dried using a lab scale forced air dehydrator (ST‐01, Septree, China). A domestic grinder (Philips, simply silent, 600w) was used to transform dried sliced vegetables into powder form. An 80‐mesh sieve was used to separate coarse powder for getting fine form. Storage bags were used individually to store fine powder at 4°C for further use. On the other hand, grocery ingredients like chili powder, refined wheat flour, egg, soybean oil, salt were purchased from the local market. Tomato paste was made from fresh tomato using a mixture jar of grinder (Philips, simply silent, 600w) by optimizing water percentage through several trial‐and‐error basis. Branded whole wheat flour was procured from a super shop to substitute refined flour at various percentages. A commercially available pasta sample was included as a commercial reference to provide a practical benchmark for comparison with the laboratory‐developed pasta formulations. The commercial product was not considered a true experimental control because its ingredient composition, processing conditions, and drying parameters could not be standardized with those of the developed formulations. Therefore, the term “commercial reference” was used throughout the study.

### 2.2 Reagents and Chemicals

Boric acid, sodium hydroxide, 37% hydrochloric acid, 98% sulfuric acid, and acetic acid were procured from Merck (New Jersey, USA). Petroleum ether (40:60), methanol (99.9%), and 100% ethanol (St. Louis, MO, USA) were supplied by an authorized dealer of Sigma‐Aldrich. Total dietary fiber assessment requires an assay kit which was provided by Megazyme in Ireland. Moreover, deionized water was also used for solution preparation.

### 2.3 Pasta Formulation

A total of six pasta formulations were developed to evaluate the effects of incorporating fiber and antioxidant enrich flours specifically green banana flour and whole wheat flour into refined wheat flour‐based pasta. These formulations were divided into two groups: WCL—pasta enriched with whole wheat flour and BCL—pasta enriched with green banana flour. Each group included three different formulations based on the increasing proportion of whole wheat flour (WWF) and green banana flour (GBF). The formulation pattern was designed for a 500.0 g sample and composed of the following ingredients: WWF, GBF, refined wheat flour, cauliflower, onion powder, garlic powder, tomato paste, salt, chili powder, and egg. Egg was incorporated because it is a traditional ingredient commonly used in fresh pasta formulations to improve dough cohesiveness, elasticity, texture, color, cooking stability, and overall sensory quality. The study did not employ a factorial experimental design. Instead, two independent formulation series were developed: one incorporating green banana flour (BCL) and the other incorporating whole wheat flour (WCL) at substitution levels of 10%, 15%, and 20%, with each series compared separately against the control pasta. The selected substitution levels were based on preliminary laboratory trials, which indicated that substitutions above 20% adversely affected dough handling, cooking quality, and sensory properties, whereas lower levels produced only limited nutritional improvement. Formulation pattern presented in the supplementary documents: Table S1.

### 2.4 Pasta Preparation

**FIGURE 1:**
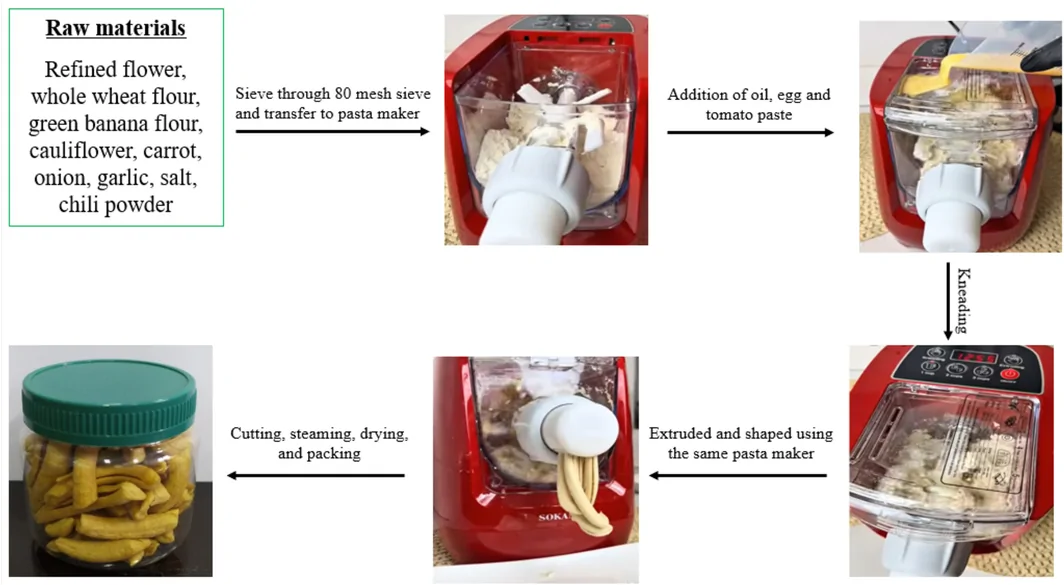
A schematic representation of experimental design.

Pasta samples were prepared according to the formulations presented in Table S1. For each formulation, the accurately weighed flour components and other dry ingredients were thoroughly mixed to ensure uniform distribution. The mixture was then combined with the predetermined amounts of tomato paste and egg and transferred to a domestic pasta maker (Model SK‐1776, Sokany, China). The ingredients were kneaded at medium speed for 5 min until a homogeneous and cohesive dough was obtained. No additional water was added, as the amount of tomato paste had been predetermined through preliminary formulation trials to provide sufficient moisture for dough development. Figure 1 presented a schematic flow chart of the experimental design.

**FIGURE 2:**
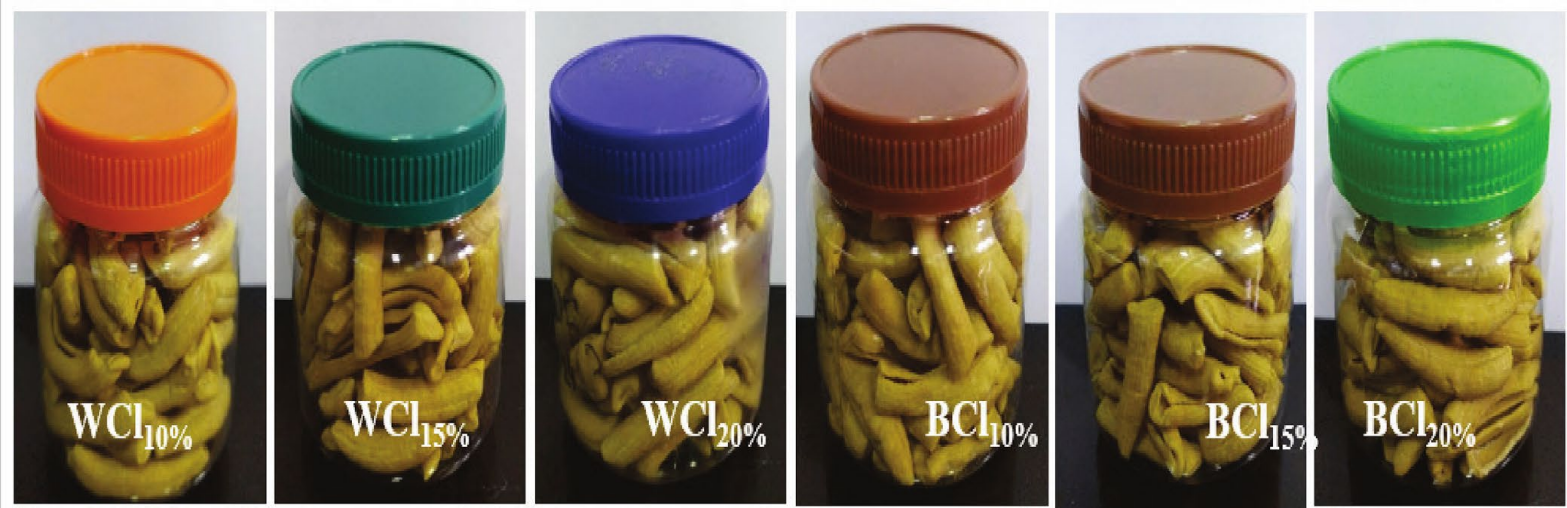
Developed pasta. WCL_10%_, WCL_15%_ and WCL_20%_: 10%, 15% and 20% whole wheat flour added pasta, respectively; BCL_10%_, BCL_15%_ and BCL_20%_: 10%, 15% and 20% green banana flour added pasta, respectively.

Following kneading, the dough was rested at room temperature for 30 min to allow moisture equilibration and dough relaxation. The rested dough was subsequently extruded and shaped using the same pasta maker under identical processing conditions for all formulations. The shaped pasta samples were steamed for 5 min and then transferred to an air‐circulation dehydrator (Model ST‐01, Septree, China). Drying was carried out at 45°C for 12 h. After drying, the pasta samples were cooled to room temperature and packed in airtight zip‐lock polyethylene bags until further physicochemical, nutritional, cooking, textural, antioxidant, and sensory analyses. Figure 2 presenting the final product of all formulations.

### 2.5 Assessment on Characteristics of Pasta Samples

#### 2.5.1 Water Activity

Water Activity Meter (Model: RS 200, Novasina AG, Switzerland) was used to measure the water activity (*a*_w_). Standard solutions (NaCl: 0.760 *a*_w_, KCl: 0.920 *a*_w_, and distilled water: 1.000 *a*_w_) were used to calibrate the device. A sample of ground pasta weighing around 3–5 g was put in a sterile sample cup and put into the chamber at about 25°C. After 5–15 min for each sample, the exhibited *a*_w_ values were recorded during measurements at a regulated temperature of 25°C. All measurements were done in triplicates for accuracy and reported the mean ± standard deviation.

#### 2.5.2 Color Characteristics

Portable Chroma Meter (CR‐400, Konica Minolta, Japan) calibrated with a standard white tile (L* = 97.63, a* = −0.48, b* = +2.17) was used to measure the color of uncooked pasta samples. Reflectance mode measurements were made using the CIELAB system (L*, a*, b*) with a 10° observer angle and D65 light. The color parameters were recorded based on the CIELAB system, where: L* indicates the degree of lightness, a* represents the red‐green axis, and b* denotes the yellow‐blue axis. To ensure accuracy, measurements were taken from three locations after the samples were evenly distributed in petri dishes. The results were then averaged and presented as mean ± standard deviation. To assess the deviation in color between treated samples and the control, the total color difference (Δ*E*) was computed using the following Equation 1.

(1) $$\Delta E={\left\{{\left(L_{1}-L_{0}\right)}^{2}+{\left(a_{1}-a_{0}\right)}^{2}+{\left(b_{1}-b_{0}\right)}^{2}\right\}}^{1/2}$$

Here, *L*_0_, *a*_0_, *b*_0_ correspond to the control sample values, and *L*_1_, *a*_1_, *b*_1_ refer to the values of the respective sample under investigation.

#### 2.5.3 Cooking Quality Attributes

Cooking time for the pasta samples was optimized according to the AACC method 66–55.01 (AACC 2005). The ideal cooking time (OCT) for every pasta sample was established in order to guarantee consistent quality assessment. Timing started when about 10 g of dried pasta was submerged in 300 mL of distilled water. One piece was taken out every 30 s for 4 min, rinsed with cold water, and then cut to view the cross‐section. When there was no whiter core, the cooking process was deemed finished. Each sample underwent the procedure three times, and the average time was noted as the OCT. Water uptake index, cooking loss, and texture profile were assessed following the Axenti et al. (2023), and Islam et al. (2025) methodology with slight modification and details procedure are provided in File S1.

### 2.6 Nutritional Profile

#### 2.6.1 Proximate Composition

Moisture, protein, fat, and ash contents were determined using standardized AOAC (2005) methods: AOAC 930.15 for moisture, AOAC 2001.11 for protein, AOAC 2003.05 for fat, and AOAC 924.05 for ash (Latimer 2005). Carbohydrate content was calculated by difference, following the guidelines provided by FAO (2003). The caloric value (kcal/100 g) was estimated using the Atwater general factors assigning 4 kcal/g for both carbohydrates and proteins, and 9 kcal/g for fats. Minerals and water‐soluble vitamins were assessed following the procedure described by Islam et al. (2024) and Rashid et al. (2024) respectively. The details are provided in the supplementary documents.

#### 2.6.2 Total Dietary Fiber Analysis

Total dietary fiber (TDF) content of pasta samples was determined using the enzymatic‐gravimetric method described in AOAC 991.43, utilizing a Foss Fibertec 1023 Fiber Analyzer (Foss Analytical, Denmark). Approximately 1.00 ± 0.005 g of defatted, moisture free ground pasta was weighed into digestion flasks. Samples underwent sequential enzymatic digestion involving heat‐stable α‐amylase (95°C for 30 min), protease (60°C for 30 min), and amyloglucosidase (60°C for 30 min at pH 4.5). Soluble fibers were precipitated using 95% ethanol (4:1, v/v), and the residue was vacuum filtered and thoroughly washed with 78% ethanol, 95% ethanol, and acetone to remove non‐fiber components. The fiber was then dried at 105°C to a constant weight, followed by ashing at 525°C for 2 h. TDF was calculated by subtracting the ash and protein content from the final residue.

#### 2.6.3 Antioxidant Activity

Phenolic compounds were extracted following the method of Gull et al. (2018) with slight modifications. Briefly, 10 g of uncooked and cooked pasta were extracted twice separately with 100 mL of 80% methanol for 2 h at 200 rpm on an orbital shaker. The combined filtrates were concentrated using a rotary evaporator (BM510, RE801, Yamato, Japan) and reconstituted to 10 mL with 100% methanol. Extracts were stored at 4°C for further analysis. Total phenolic content (TPC), total flavonoid content (TFC), and total antioxidant capacity (TAC) of uncooked and cooked pasta phenolic extracts were determined using the Folin–Ciocalteu method, aluminum chloride colorimetric assay, and phosphomolybdenum assay, respectively. The test methods explained in detail in File S1.

#### 2.6.4 DPPH• Scavenging Activity

The 1,1‐diphenyl‐2‐picrylhydrazyl (DPPH) free radical scavenging activity of uncooked and cooked pasta samples was evaluated with slight modifications following the method of Gull et al. (2018). In this assay, 100 μL of sample or ascorbic acid standard at concentrations of 100 μg/mL was mixed with 3.0 mL of 0.1 mM DPPH solution prepared in methanol. The reaction mixture was incubated in the dark at room temperature for 30 min. Absorbance was measured at 517 nm against a methanol blank, with the DPPH solution alone serving as the control. The percentage of DPPH radical scavenging activity was calculated using the appropriate (Equation 2).

(2) $$\text{Inhibition}\;\left(\%\right)=\left[\;1-\frac{\text{Absorbane of sample}}{\text{Absorbance of control}}\;\right]\times 100$$

#### 2.6.5 ABTS•^+^ Scavenging Activity

The ABTS●^+^ radical scavenging activity of uncooked and cooked pasta phenolic extracts was assessed using a modified version of the method described by Xiao et al. (2020). A volume of 100 μL of sample or ascorbic acid standard 100 μg/mL was added to 4.0 mL of ABTS●^+^ working solution prepared in phosphate buffer (pH 7.4), followed by incubation in the dark at room temperature for 30 min. Absorbance was recorded at 734 nm using phosphate buffer as the blank, with the ABTS●^+^ solution alone serving as the control. Radical scavenging activity was calculated using the corresponding (Equation 2).

### 2.7 Sensory Attributes

Sensory evaluation of the prepared pasta was conducted following the method described by Sarker et al. (2025) with slight modifications. The cooked pasta samples were first coded with random numbers and served to the panelists in a randomized order within 5 min after cooking to avoid quality deterioration. A total of 10 trained panelists, comprising 6 female and 4 male members, selected from the sensory panel of the Institute of Food Science and Technology (IFST), Bangladesh Council of Scientific and Industrial Research (BCSIR), Dhaka, Bangladesh, participated in the evaluation. Each panelist was seated in an individual sensory booth to ensure unbiased assessment. The coded samples were distributed by the panel leader for identification purposes only. A structured sensory evaluation form was provided to each participant, which included attributes such as color, texture, flavor, taste, and overall acceptability. In addition, panelists were asked to rate overall acceptability separately using the same form. The sensory attributes were evaluated using a 9‐point hedonic scale, where 9 indicated “like extremely” and 1 indicated “dislike extremely.” All panelists provided written informed consent prior to participation in the evaluation process. The collected data were compiled and statistically analyzed, and the panel leader prepared the final report presenting the results as mean ± standard deviation (SD) for each sensory attribute.

### 2.8 Statistical Analysis

All measurements were performed in triplicate unless otherwise specified, and results were reported as mean ± standard deviation (SD). Statistical analysis was conducted using one‐way analysis of variance (ANOVA), followed by Tukey's post hoc test to determine significant differences among sample means at *p* ≤ 0.05. Data analysis was performed using OriginPro 2024 software (OriginLab Corporation, Northampton, USA).

## 3 Results and Discussion

### 3.1 Water Activity

**FIGURE 3:**
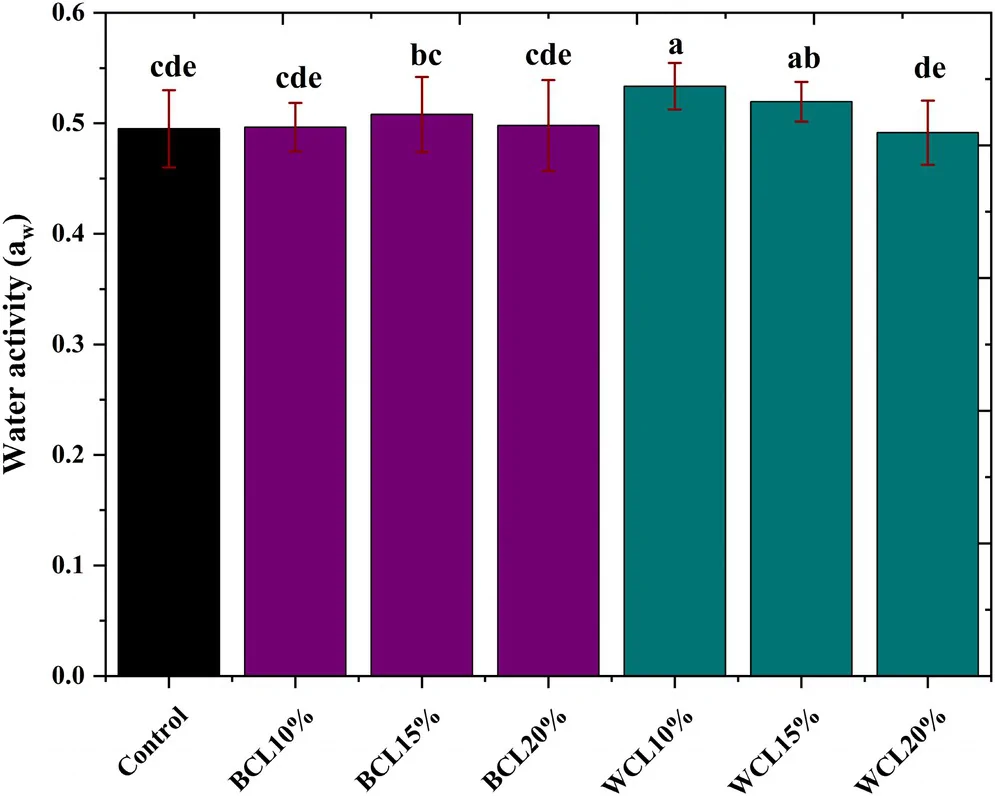
Water activity of fiber and antioxidant enriched formulated pasta. Values are expressed as mean ± standard deviation (*n* = 3). Different letters indicate significant differences determined by Tukey's test (*p* ≤ 0.05) in the mean value.

Water activity (*a*_w_) is a critical factor in food processing, as it influences microbial stability, shelf life, and packaging requirements (Lee and Robertson 2022). Defined as the ratio of the vapor pressure of water in a food product to that of pure water at the same temperature, *a*_w_ values range from 0 (completely dry) to 1.0 (pure water). Unlike total moisture content, *a*_w_ more directly affects microbial proliferation and both enzymatic and non‐enzymatic reactions (Barbosa‐Canovas et al. 2021). Figure 3 illustrates the *a*_w_ values of the pasta samples. The control pasta exhibited an *a*_w_ of approximately 0.49, which is well within the microbiologically safe range for dried foods (*a*_w_ < 0.60). Among the formulations, BCL_15%_ (0.52) and WCL_10%_ (0.55) showed significantly higher *a*_w_ values (*p* ≤ 0.05), indicating that the incorporation of green banana and whole wheat flours increased the retention of free water in the pasta matrix. This effect is likely attributed to the hydrophilic properties of dietary fibers and pectic substances present in the added flours, which enhance water binding capacity and reduce water mobility (Ovando‐Martínez et al. 2009). Despite these increases, all samples maintained *a*_w_ values below 0.60, supporting their microbial stability and potential for extended shelf life (Fennema 1996). Notably, WCL_10%_ and WCL_15%_ recorded the highest *a*_w_ levels (0.55 and 0.53, respectively), both significantly higher than the control, yet still within safe storage limits. These findings suggest that enrichment with fiber and antioxidant enrich ingredients can enhance moisture retention without compromising product safety or shelf stability.

### 3.2 Color Characteristics

Table 1 presents the color attributes (L*, a*, b*, and ∆*E*) of developed pasta samples. The lightness (L*) values of all enriched formulations (80.23–83.50) were not significantly different (*p* > 0.05) from the control (83.83), indicating that fiber enrichment had minimal impact on pasta brightness. However, the a* values increased significantly (*p* ≤ 0.05) in all BCL and WCL samples (3.66–4.41), compared to the negative a* value of the control (−5.47), reflecting a noticeable shift toward redness. Similarly, b* values significantly decreased (*p* ≤ 0.05) in all enriched samples (24.85–26.96) relative to the control (38.50), suggesting reduced yellowness due to the replacement of refined flour with less pigmented flours. The total color difference (∆*E*) values also varied significantly (*p* ≤ 0.05), with enriched samples ranging from 12.66 to 15.28, confirming perceptible differences in appearance compared to the control (∆*E* = 34.68). Among the samples, WCL_20%_ exhibited the smallest overall color shift (∆*E* = 12.66). These results are consistent with earlier findings that incorporation of green banana and whole wheat flours alters pasta color by introducing non‐pigmented dietary fibers and phenolic compounds that interact with starch‐protein matrices (Karaman et al. 2025; Ovando‐Martínez et al. 2009). Despite these changes, all color parameters remained within a range considered acceptable for consumer products.

**TABLE 1:** Comparative color characteristics data of the green banana and whole wheat flour enriched pasta with control pasta.

Test parameters	Formulation						
	Control	BCL_10%_	BCL_15%_	BCL_20%_	WCL_10%_	WCL_15%_	WCL_20%_
L	83.83 ± 2.25a	81.41 ± 0.22a	80.48 ± 0.33a	80.23 ± 0.30a	83.50 ± 0.30a	82.74 ± 0.67a	81.85 ± 0.64a
A	−5.47 ± 0.16a	3.66 ± 0.08b	4.41 ± 0.09b	4.13 ± 0.03b	3.66 ± 0.08b	4.41 ± 0.09b	4.13 ± 0.03b
B	38.50 ± 1.07a	25.70 ± 0.32b	26.96 ± 0.16b	25.44 ± 0.16b	26.15 ± 0.20b	26.76 ± 0.14b	24.85 ± 0.08b
ΔE	34.68 ± 0.20a	13.30 ± 0.14b	15.28 ± 0.24d	13.65 ± 0.21e	13.75 ± 0.21d	14.22 ± 0.05c	12.66 ± 0.17e

*Note:* Results are expressed as mean ± standard deviation (*n* = 3). Different superscript letters in the same row indicate significant differences determined by Tukey's test (*p* ≤ 0.05) in the mean value. Control: Market products.

### 3.3 Cooking Characteristics

The cooking performance of pasta enriched with green banana and whole wheat flours, in comparison to the control, is detailed in Table 2. The optimum cooking time (OCT) for enriched samples ranged from 10.12 to 10.81 min, showing no significant difference (*p* > 0.05) compared to the control (9.03 min), which aligns with previous studies where fiber‐enriched pasta exhibited marginally increased or comparable cooking times (Islam et al. 2025). Water uptake index (WUI) values increased significantly (*p* ≤ 0.05) across all formulations (104.69–111.06) relative to the control (100.18), indicating enhanced moisture absorption due to the hydrophilic nature of dietary fibers (Sempio et al. 2025). Similarly, cooking loss (CL) was significantly higher (*p* ≤ 0.05) in enriched samples (8.97%–10.86%) compared to the control (8.85%), reflecting a less cohesive starch‐protein matrix that allows greater leaching of solids during cooking. These findings are consistent with reports that incorporation of high‐fiber ingredients like banana flour or oat husks increases water retention and cooking loss due to interference with gluten network formation (Biernacka et al. 2023; Moo‐Huchin et al. 2025). The results suggest that, while fiber enrichment enhances water absorption a desirable trait, it may compromise cooking integrity through elevated solute loss, reinforcing the need for formulation optimization to balance functional and sensory quality.

**TABLE 2:** Comparative cooking characteristics data of the green banana and whole wheat flour enriched pasta with control pasta.

Test parameters	Formulation						
	Control	BCL_10%_	BCL_15%_	BCL_20%_	WCL_10%_	WCL_15%_	WCL_20%_
OCT (min)	9.03 ± 0.53a	10.12 ± 042a	10.64 ± 0.37a	10.46 ± 0.59a	10.67 ± 0.85a	10.81 ± 0.62a	10.81 ± 0.62a
WUI (%)	100.18 ± 2.82a	108.28 ± 1.99b	108.96 ± 1.99b	111.06 ± 5.62b	107.24 ± 2.11b	106.89 ± 2.04b	104.69 ± 5.43b
CL (%)	8.85 ± 0.41a	8.97 ± 0.43a	9.91 ± 0.57b	10.14 ± 0.37b	10.45 ± 0.55b	10.79 ± 0.59bc	10.86 ± 0.49c

*Note:* Results are expressed as mean ± standard deviation (*n* = 2). Different superscript letters in the same row indicate significant differences determined by Tukey's test (*p* ≤ 0.05) in the mean value. Control: Market products.

Abbreviations: CL, cooking loss; OCT, optimum cooking time; WUI, water uptake index.

### 3.4 Texture Profile

**FIGURE 4:**
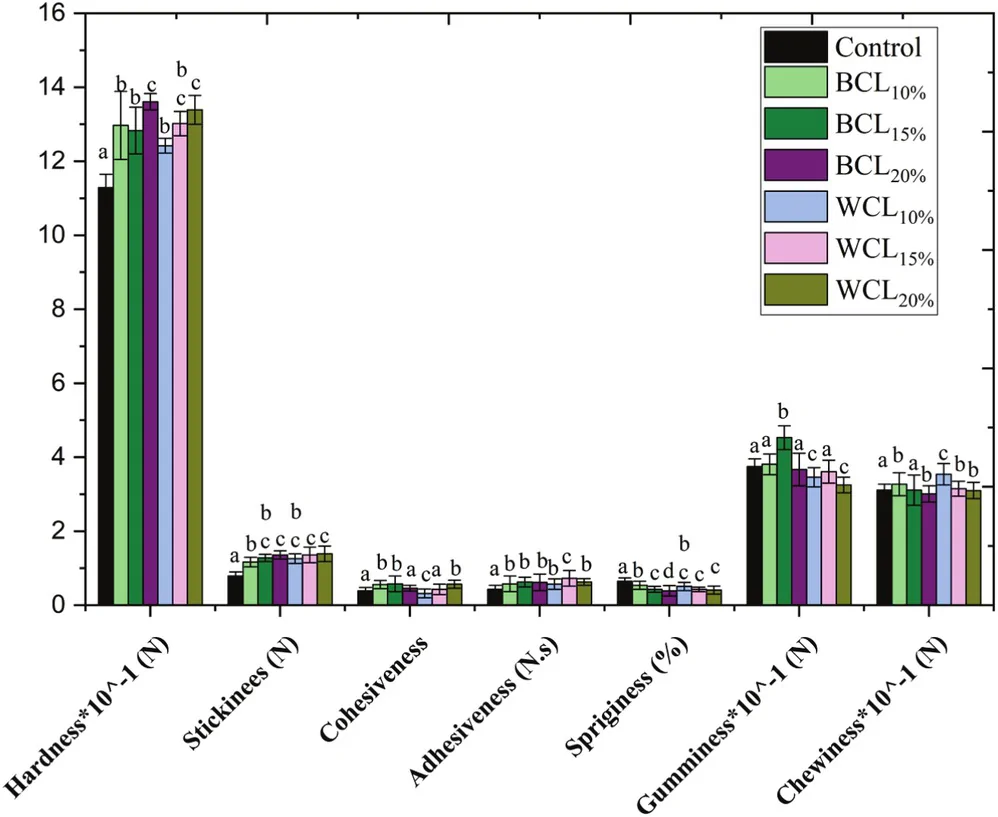
Texture analysis data of cooked pasta products. Values are expressed as mean ± standard deviation (*n* = 3). Different letters within same test parameters indicate significant differences determined by Tukey's test (*p* ≤ 0.05) in the mean value.

The texture profile analysis of cooked pasta revealed notable variations across key parameters (all values ×10−1 N except where specified) (Figure 4). Hardness increased significantly in enriched samples, rising from 11.29 N in the control to values up to 13.61 N in BCL_20%_, indicating firmer texture likely resulting from increased fiber and disrupted starch‐protein matrix (*p* ≤ 0.05). Stickiness also rose markedly from 0.79 N to between 1.17 N and 1.39 N suggesting increased surface residue during mastication. Cohesiveness improved in most formulations (0.56–0.58) compared to the control (0.39), implying stronger internal bonding. Adhesiveness increased (0.43 to ~0.63–0.73 N·s), reflecting greater sample‐probe interaction during texture testing. Springiness decreased in enriched pasta (0.39%–0.54% vs. 0.65% in control), suggesting reduced elasticity. Gumminess and chewiness largely paralleled hardness and cohesiveness trends; gumminess ranged from 3.25 to 4.53 N, while chewiness ranged from 3.01 to 3.54 N. These patterns are consistent with previous findings showing that pasta enriched with fiber sources tends to exhibit increased firmness, adhesiveness, and reduced springiness due to gluten network interference (Krishnan et al. 2012; Adiba et al. 2024). Texture profile analysis parameters effectively captured these texture shifts, reflecting altered mouthfeel and mechanical structure following enrichment with dietary fibers.

### 3.5 Nutritional Assessment

Pasta proximate composition of samples showed statistically significant differences (*p* ≤ 0.05) compared to the control (Table 3). Moisture content increased significantly in enriched formulations (8.32–9.99 g/100 g) from 7.95 g/100 g in the control, indicating enhanced water retention likely due to hydrophilic fiber components (Mishra et al. 2024). Ash content nearly tripled from 0.41 g/100 g in the control to as high as 1.39 g/100 g in WCL_20%_, highlighting the mineral‐rich nature of the added flours (Bustos et al. 2011). Fat content also rose significantly in BCL formulations (up to 12.60 g/100 g), consistent with increased lipid‐bound phenolics or oil‐bearing residues (Bustos et al. 2011). Protein content showed modest increases (13.85 to 16.12 g/100 g), reflecting additive contributions from whole grain and banana matrices. Carbohydrate levels significantly decreased (62.77–76.91 g/100 g), which can be attributed to substitution with non‐digestible fiber fractions (Mishra et al. 2024). Consequently, caloric values increased for some enriched samples (up to 422 kcal/100 g in BCL_15%_) before declining in higher whole‐wheat additions, due to shifts in macronutrient balance. These trends align with broader observations that fiber enrichment typically reduces carbohydrate content while boosting ash and moisture, without substantially affecting protein levels, thereby enhancing nutritional profile without sacrificing product acceptability (Mishra et al. 2024; Bustos et al. 2011).

**TABLE 3:** Nutritional data of fiber enrich formulated pasta.

Test parameters	Formulation						
	Control	BCL_10%_	BCL_15%_	BCL_20%_	WCL_10%_	WCL_15%_	WCL_20%_
Moisture (g/100 g)	7.95 ± 0.07a	8.60 ± 0.28b	8.98 ± 0.30bc	8.32 ± 0.48b	9.99 ± 0.23c	9.13 ± 0.63cb	9.81 ± 0.78c
Ash (g/100 g)	0.41 ± 0.02a	0.82 ± 0.02b	1.19 ± 0.08 cd	1.25 ± 0.04 cd	1.00 ± 0.04cb	1.11 ± 0.05c	1.39 ± 0.04d
Fat (g/100 g)	0.89 ± 0.03a	8.59 ± 0.53b	12.60 ± 0.78c	11.03 ± 0.76c	9.82 ± 0.59b	8.00 ± 0.80bd	7.17 ± 0.08d
Protein (g/100 g)	13.85 ± 0.49a	14.50 ± 0.85ab	14.46 ± 0.63ab	14.25 ± 0.64ab	15.07 ± 0.36b	15.17 ± 0.86b	16.12 ± 1.22b
Carbohydrate (g/100 g)	76.91 ± 0.42a	67.49 ± 1.69b	62.77 ± 1.79c	64.16 ± 0.88bc	65.92 ± 0.41bc	67.59 ± 0.98bc	65.51 ± 2.13bc
Energy (kcal/100 g)	371.03 ± 0.0a	405.23 ± 1.44b	422.28 ± 2.36c	416.89 ± 5.88c	405.12 ± 2.2b	398.98 ± 6.7bd	391.03 ± 2.89d
Na (mg/100 g)	43.42 ± 3.09a	80.95 ± 9.67b	82.33 ± 4.81b	80.53 ± 5.85b	82.70 ± 3.66b	70.77 ± 3.92c	95.67 ± 3.83d
Mg (mg/100 g)	2.28 ± 0.01a	19.10 ± 0.10b	21.95 ± 0.96bc	25.89 ± 1.21c	18.67 ± 0.51b	24.26 ± 0.34c	26.14 ± 1.13c
P (mg/100 g)	30.67 ± 1.58a	71.17 ± 2.44b	72.87 ± 0.83bc	77.75 ± 1.44c	87.05 ± 4.02d	108.18 ± 3.13e	107.85 ± 2.40e
K (mg/100 g)	224.36 ± 7.6a	450.27 ± 16.1b	456.98 ± 14.0b	426.65 ± 19.9c	397.26 ± 7.9d	379.20 ± 17.d	437.45 ± 27.0bc
Ca (mg/100 g)	172.56 ± 2.9a	251.65 ± 5.17b	260.94 ± 4.35b	261.94 ± 2.99b	234.34 ± 1.9b	242.39 ± 1.8b	292.86 ± 15.7c
Fe (mg/100 g)	2.71 ± 0.25a	13.23 ± 0.28b	13.53 ± 0.19b	14.48 ± 0.59bc	15.21 ± 0.74c	15.34 ± 0.33 cd	17.08 ± 0.23d
Cu (mg/100 g)	0.37 ± 0.02a	2.32 ± 0.17b	2.94 ± 0.16c	2.76 ± 0.07bc	3.40 ± 0.21d	3.72 ± 0.15e	3.75 ± 0.67e
Zn (mg/100 g)	0.15 ± 0.03a	0.61 ± 0.01b	0.56 ± 0.00b	0.81 ± 0.02c	0.76 ± 0.04c	0.92 ± 0.04d	0.92 ± 0.04d

*Note:* Results are expressed as mean ± standard deviation (*n* = 2). Different superscript letters in the same row indicate significant differences determined by Tukey's test (*p* ≤ 0.05) in the mean value. Control: Market products.

The mineral composition showed a significant improvement compared to the control sample (*p* ≤ 0.05), confirming the nutritional benefits of incorporating these fiber‐rich ingredients. Sodium content increased from 43.42 mg/100 g in the control to 95.67 mg/100 g in WCL_20%_, which may be attributed to the inherent mineral content of the added flours as well as enhanced salt retention during formulation. A substantial rise in magnesium was observed, ranging from 2.28 mg/100 g in the control to 26.14 mg/100 g in WCL_20%_, reflecting the magnesium‐rich nature of both green banana and whole wheat. This aligns with previous findings by Dziki and Gawlik‐Dziki (2021), who noted similar improvements in mineral composition when wheat bran was used in pasta enrichment. Phosphorus content also increased significantly, from 30.67 mg/100 g in the control to 108.18 mg/100 g in WCL_15%_, suggesting that both enrichment ingredients are effective sources of bioavailable phosphorus. The most pronounced increase was observed in potassium, which rose from 224.36 mg/100 g to 456.98 mg/100 g in BCL_15%_, consistent with potassium's high concentration in green banana. Calcium, another vital mineral, increased from 172.56 mg/100 g in the control to 292.86 mg/100 g in WCL_20%_, enhancing the functional food potential of the enriched pasta. In terms of trace elements, iron levels improved substantially, with the highest concentration (17.08 mg/100 g) found in WCL_20%_, a nearly six‐fold increase over the control, indicating that enrichment could help address iron deficiency. Similarly, copper content increased from 0.37 mg/100 g in the control to 3.75 mg/100 g in WCL_20%_, and zinc content rose from 0.15 mg/100 g to 0.92 mg/100 g in both WCL_15%_ and WCL_20%_ formulations. These enhancements reflect the mineral dense nature of the incorporated ingredients and confirm earlier findings by Islam et al. (2025), who demonstrated improved mineral content in fiber and antioxidant enriched pasta.

### 3.6 Assessment of B Vitamins

**FIGURE 5:**
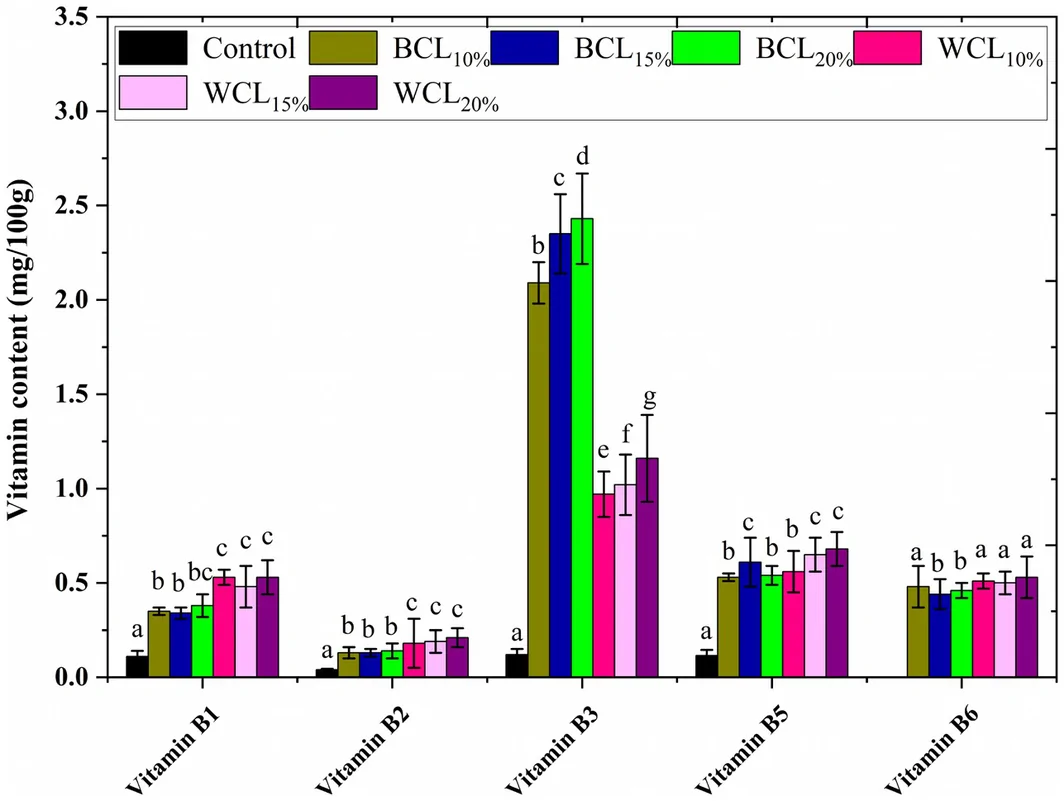
Water soluble vitamin data of uncooked pasta products. Values are expressed as mean ± standard deviation (*n* = 2). Different letters within same test parameters indicate significant differences determined by Tukey's test (*p* ≤ 0.05) in the mean value.

Figure 5 presents the concentrations of water‐soluble B vitamins (B1, B2, B3, B5, and B6) in control and enriched uncooked pasta samples. The enrichment with green banana and whole wheat flour resulted in significant increases (*p* ≤ 0.05) in the vitamin content compared to the control, demonstrating the functional benefit of incorporating these natural sources. Notably, Vitamin B3 (niacin) levels were the highest among all measured vitamins. Control pasta had the lowest concentration (~0.25 mg/100 g), whereas BCL_20%_ showed the peak value (2.9 mg/100 g), followed by BCL_15%_ and WCL_20%_. The progressive increase suggests that green banana flour, particularly at higher concentrations, is a rich source of niacin. These results are in line with findings by Castelo‐Branco et al. (2017), who reported that green banana pulp contributes significantly to the B3 pool in functional food applications. Vitamin B1 content also increased with enrichment from 0.2 mg/100 g in the control to 0.5–0.6 mg/100 g in the WCL and BCL variants, particularly in WCL_20%_. Similarly, Vitamin B2 improved significantly in the enriched samples, though the absolute values remained modest (0.3–0.4 mg/100 g). These enhancements support prior observations by Conti et al. (2023), who documented improved thiamine and riboflavin retention in pasta enriched with natural fiber‐rich components. Vitamin B5 content ranged from 0.4 mg/100 g in the control to 0.7 mg/100 g in enriched variants, particularly BCL_15%_, WCL_15%_, and WCL_20%_, suggesting both banana and whole wheat flours contribute to improved vitamin bioavailability. Vitamin B6, while relatively stable across samples, also showed statistically significant increases in most enriched formulations (except WCL_20%_), again reflecting the nutritional advantage of using antioxidant‐rich plant ingredients. Overall, these findings indicate that the enrichment of pasta with green banana and whole wheat flours significantly boosts its B vitamin profile. This enhancement is nutritionally important given that B vitamins are essential for energy metabolism, neurological function, and red blood cell formation (Sempio et al. 2025). Thus, fiber and antioxidant source enrichment not only improves pasta's functional quality but also contributes meaningfully to micronutrient intake.

### 3.7 Total Dietary Fiber

**FIGURE 6:**
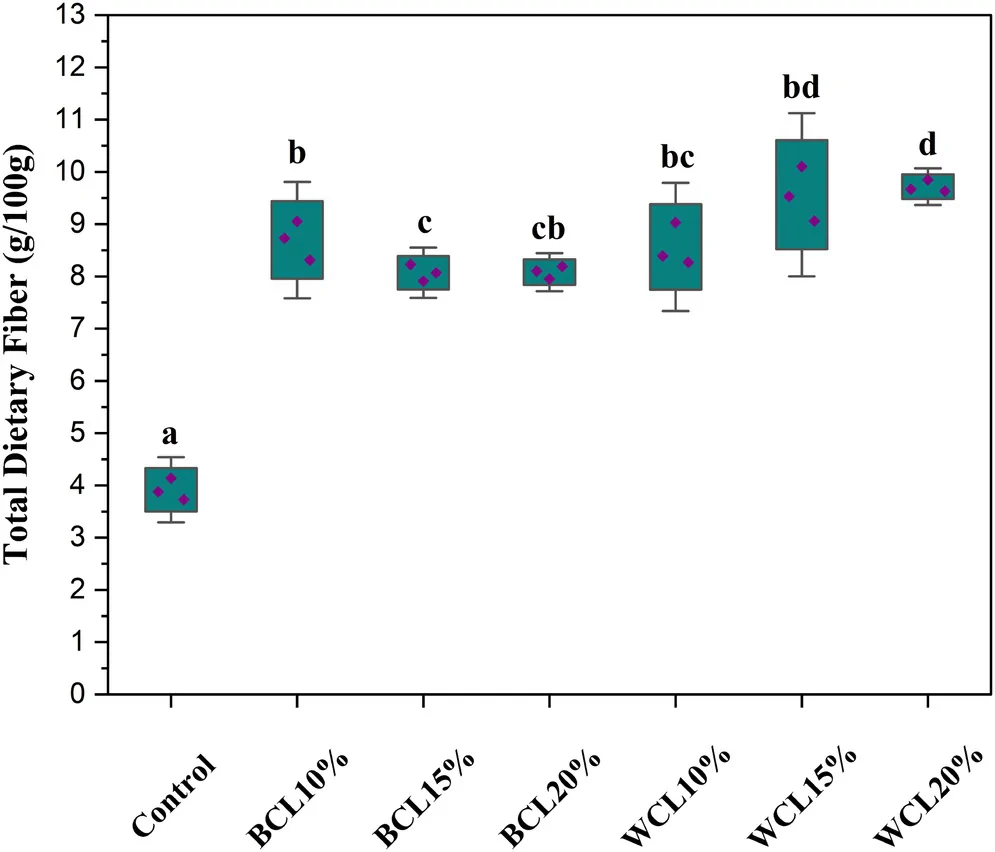
Total dietary content of uncooked pasta products. Values are expressed as mean ± standard deviation (*n* = 3). Different letters within same test parameters indicate significant differences determined by Tukey's test (*p* ≤ 0.05) in the mean value.

Figure 6 illustrates the Total Dietary Fiber (TDF) content in formulated pasta compared to a control. The control pasta exhibited the lowest TDF content (4.2 ± 0.5 g/100 g), significantly lower (*p* ≤ 0.05) than all enriched variants. Among the enriched samples, BCL_10%_ and WCL_15%_ had the highest TDF contents, reaching 9.5 ± 0.7 g/100 g and 9.7 ± 1.0 g/100 g, respectively, more than doubling the fiber level of the control. The TDF content in BCL_15%_ and BCL_20%_ was 8.3 ± 0.4 g/100 g and 8.4 ± 0.5 g/100 g, respectively. Although still significantly higher than the control (*p* < 0.05), these values were lower than BCL_10%_. A similar pattern was observed in the WCL series: WCL_10%_ yielded 9.1 ± 1.1 g/100 g, while WCL_20%_ had 8.6 ± 0.3 g/100 g. The slightly reduced values in higher inclusion levels may be attributed to matrix saturation or interaction effects between starch, protein, and fiber, as previously reported by Suzauddula et al. (2021) and Laleg et al. (2016). The enhanced dietary fiber content in the enriched pasta samples is primarily due to the high fiber concentrations of green banana and whole wheat flour, which are rich in resistant starch, hemicellulose, and lignin (Ovando‐Martínez et al. 2009). These improvements are nutritionally significant, as increasing dietary fiber intake is associated with reduced risk of non‐communicable diseases, improved gut health, and better glycemic control (McKeown et al. 2022). According to the Codex Alimentarius guidelines, foods containing more than 6 g of fiber per 100 g can be labeled as “high in fiber,” which all enriched samples in this study exceed, further reinforcing their functional food value.

### 3.8 Antioxidant Activity

Enrichment of pasta with green banana and whole wheat flour significantly (*p* ≤ 0.05) enhanced the phenolic, flavonoid, and antioxidant profiles compared to the control (Table 4). TPC increased markedly before cooking, from 2.16 ± 1.04 μg gallic acid equivalent (GAE)/g dry weight (DW) in the control to 27.78 ± 2.55 μg GAE/g DW in BCL_20%_. After cooking, some loss was observed, yet enriched pasta still retained higher values (WCL_20%_ = 11.77 ± 2.23) compared to the control (1.13 ± 0.82). Similar patterns have been reported, as thermal processing often reduces free phenolics but maintains bound phenolics that continue to contribute to antioxidant activity (Fares et al. 2010).

**TABLE 4:** Total phenolic, total flavonoid, total antioxidant, DPPH and ABTS data of formulated pasta.

Test parameters	Formulation						
	Control	BCL_10%_	BCL_15%_	BCL_20%_	WCL_10%_	WCL_15%_	WCL_20%_
TPC_BC (μg GAE/g DW)	2.16 ± 1.04a	26.14 ± 2.52b	23.87 ± 2.48bc	27.78 ± 2.55b	21.46 ± 2.42d	24.85 ± 2.50cb	24.99 ± 1.49c
TPC_AC (μg GAE/g DW)	1.13 ± 0.82a	15.03 ± 1.10b	15.37 ± 2.11b	15.53 ± 2.11b	10.15 ± 2.20c	10.54 ± 1.21c	11.77 ± 2.23c
TFC_BC (μg QE/g DW)	1.02 ± 0.11a	37.74 ± 2.37b	35.25 ± 2.35bc	34.31 ± 2.03c	35.84 ± 2.84b	39.50 ± 3.39bd	40.17 ± 2.40bd
TFC_AC (μg QE/g DW)	1.12 ± 0.04a	69.92 ± 2.69b	78.58 ± 2.78c	75.44 ± 2.75c	91.04 ± 2.91d	90.77 ± 3.91d	85.93 ± 2.89e
TAC_BC (μg AAE/g DW)	6.23 ± 1.19a	57.38 ± 2.15b	58.60 ± 1.17b	59.56 ± 2.91b	81.83 ± 2.66c	81.58 ± 3.61c	84.55 ± 2.09c
TAC_AC (μg AAE/g DW)	4.95 ± 0.60a	71.49 ± 2.29b	63.06 ± 2.61c	62.19 ± 3.37c	68.24 ± 3.47d	72.48 ± 2.95b	69.61 ± 2.32bd
DPPH_BC (% inhibition)	13.42 ± 1.19a	80.95 ± 2.63b	82.33 ± 2.85b	80.53 ± 2.80b	82.70 ± 2.36b	90.77 ± 2.39c	95.67 ± 2.83d
DPPH_AC (% inhibition)	2.28 ± 0.05a	39.10 ± 0.11b	41.95 ± 0.86bc	35.89 ± 1.25c	38.67 ± 1.25b	44.26 ± 1.44c	46.14 ± 1.31c
ABTS_BC (% inhibition)	21.67 ± 1.53a	71.17 ± 2.14b	72.87 ± 3.07bc	77.75 ± 2.08c	77.05 ± 4.10d	78.18 ± 3.13e	77.85 ± 2.40e
ABTS_AC (% inhibition)	4.36 ± 0.36a	50.27 ± 2.11b	46.28 ± 4.06b	46.15 ± 3.43c	47.11 ± 2.13d	48.22 ± 3.21d	47.41 ± 2.21bc

*Note:* Results are expressed as mean ± standard deviation (*n* = 2). Different superscript letters in the same row indicate significant differences determined by Tukey's test (*p* ≤ 0.05) in the mean value.

Abbreviations: AAE, ascorbic acid equivalent; ABTS, 2, 2′‐azino‐bis‐(3‐ethylbenzothiazoline‐6‐sulfonic acid); AC, after cooking; BC, before cooking; DPPH, 1,1‐diphenyl‐2‐picrylhydrazyl; GAE, gallic acid equivalent; QE, quercetin equivalent; TAC, total antioxidant content; TFC, total flavonoid content; TPC, total phenolic content.

TFC showed the most striking improvement. Before cooking, values increased from 1.02 ± 0.11 μg QE/g DW in the control to 40.17 ± 2.40 in WCL_20%_. After cooking, flavonoid content was even higher, peaking at 91.04 ± 2.91 in WCL_10%_. This suggests thermal processing may enhance flavonoid extractability, consistent with earlier findings where heating disrupted plant cell matrices, releasing bound flavonoids (Zhang et al. 2022).

TAC followed a similar trend. Prior to cooking, enriched samples such as WCL_20%_ recorded 84.55 ± 2.09 μg AAE/g DW, a substantial increase over the control (6.23 ± 1.19). Post‐cooking, TAC values slightly declined but remained significantly higher than control (BCL_10%_ = 71.49 ± 2.29, compared to 4.95 ± 0.60 in control). These results align with Dziki and Gawlik‐Dziki (2021), who observed enhanced antioxidant retention in fiber‐enriched pasta due to synergistic effects between phenolics and dietary fiber.

Free radical scavenging activity also reflected these improvements. Before cooking, DPPH inhibition rose sharply from 13.42% in control to 95.67% in WCL_20%_, while post‐cooking inhibition remained high, with WCL_20%_ at 46.14% versus control at 2.28%. Likewise, before cooking, ABTS inhibition improved from 21.67% in control to 78.18% in WCL_15%_, with post‐cooking values sustaining higher levels (48.22%) than control (4.36%). The strong radical scavenging potential underscores the contribution of phenolic compounds and flavonoids from banana and whole wheat flour, confirming previous findings where fruit or vegetable‐based enrichment boosted antioxidant stability in pasta (Panza et al. 2023). The data demonstrate that incorporating banana and whole wheat flour substantially enhances pasta's phytochemical and antioxidant potential, even after cooking. This indicates that functional pasta formulations could serve as an effective vehicle for delivering bioactive compounds in the human diet.

### 3.9 Sensory Evaluation

**FIGURE 7:**
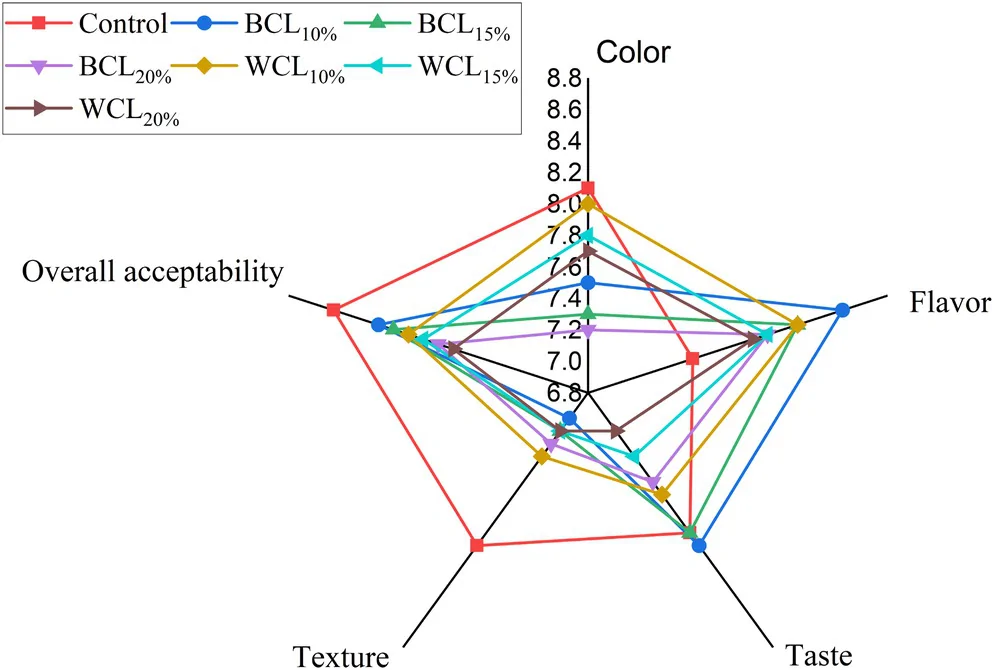
Sensory evaluation data of cooked pasta products.

The sensory evaluation of pasta enriched with green banana and whole wheat flour, compared with the control, is presented in the radar plot, covering five parameters: color, flavor, taste, texture, and overall acceptability, assessed on a 9‐point hedonic scale (Figure 7). Color was most preferred in the control (8.2) and WCL_10%_ (8.0), suggesting that moderate substitution levels did not compromise visual appeal, whereas higher substitutions such as BCL_20%_ (7.4) and WCL_20%_ (7.6) were rated significantly lower (*p* ≤ 0.05), likely due to darker pigmentation from fiber‐rich flours. This aligns with previous studies reporting that fiber addition alters lightness and yellowness in pasta, which can negatively influence sensory evaluation (Panza et al. 2023). Flavor ratings peaked in BCL_10%_ (8.2), surpassing the control (7.2), indicating that green banana flour contributed desirable sensory notes, possibly due to natural sugars and volatile compounds, while excessive substitution (7.0 in BCL_20%_ and 7.2 in WCL_20%_) reduced flavor scores, consistent with findings that higher fiber inclusion can mask or dilute characteristic pasta flavors (Zhang et al. 2022). Taste scores showed less variability, clustering between 6.8 and 7.4, with BCL_10%_ performing best, suggesting that moderate enrichment enhanced palatability without introducing off‐flavors. Texture, however, showed a reverse trend; the control pasta scored highest (7.8), whereas enriched formulations scored significantly lower (6.8–7.2), reflecting the impact of fiber on gluten network formation and starch gelatinization, leading to firmer or less elastic pasta structures (Kaur et al. 2023). Despite this, overall acceptability was not severely compromised, as both the control and BCL_10%_ achieved the highest ratings (7.8 and 8.0 respectively). In contrast, formulations with 20% substitution, particularly WCL_20%_ and BCL_20%_, recorded the lowest overall acceptability (7.0), indicating that while nutritional benefits were enhanced, sensory quality was partially sacrificed at higher inclusion levels. These findings highlight a critical trade‐off between nutritional fortification and consumer sensory acceptance. Similar observations were reported by Dziki (2021), where pasta enriched with plant‐based flours showed improved functional properties but reduced sensory scores at high substitution levels.

## 4 Conclusion

This study demonstrated that enrichment of pasta with green banana flour and whole wheat flour significantly improved its nutritional and functional profile while maintaining consumer acceptability. Proximate analysis showed notable increases in key nutrients, with protein increasing from 13.85 g/100 g in the control to 16.12 g/100 g in WCL_20%_, and dietary fiber content more than doubling, from 3.9 g/100 g in the control to 10.1–11.2 g/100 g in enriched formulations. Mineral composition also improved substantially, with calcium increasing from 172.6 mg/100 g (control) to 292.9 mg/100 g in WCL_20%_, potassium from 224.4 mg/100 g to 456.9 mg/100 g in BCL_15%_, and iron from 2.7 mg/100 g to 17.1 mg/100 g in WCL_20%_. Antioxidant analysis confirmed that enriched pasta contained markedly higher bioactive compounds, with total phenolic content increasing from 2.16 μg GAE/g DW in the control to 27.8 μg GAE/g DW in BCL_20%_, and DPPH inhibition improving from 13.4% (control) to 95.7% in WCL_20%_. Despite slight reductions after cooking, enriched formulations retained significantly higher antioxidant activity than the control. Texture analysis revealed an increase in hardness from 1.13 N in the control to 1.36 N in BCL_20%_, accompanied by higher stickiness and adhesiveness, consistent with fiber‐induced gluten dilution. Importantly, water activity values (0.49–0.55) remained below 0.60, indicating microbiological stability. Sensory evaluation highlighted 10%–15% enrichment levels as optimal, balancing enhanced nutritional and antioxidant benefits with favorable color, flavor, texture, and overall acceptability. These findings confirm the potential of banana and whole wheat flours as functional ingredients for developing nutrient‐dense antioxidant‐rich pasta.

## 5 Limitation

Although this study demonstrated the nutritional and functional potential of pasta enriched with green banana and whole wheat flours, certain limitations should be noted. The use of commercial pasta as a reference is a limitation because differences in formulation and processing may influence the observed results. Therefore, comparisons should be considered practical benchmarking rather than controlled experimental comparisons. Future studies should include a laboratory‐prepared control under identical processing conditions. The sample size for sensory evaluation was relatively small (*n* = 10), which may not fully represent broader consumer preferences. We also acknowledge that statistical assumptions such as normality and homogeneity of variance were not formally assessed. Antioxidant activity was assessed using in vitro assays, which may not directly translate to in vivo bioavailability. Additionally, storage stability and shelf‐life beyond water activity measurements were not evaluated. Future studies should include larger‐scale sensory trials, in vivo digestibility assessments, and extended shelf‐life evaluations.

## Author Contributions

**Nusrat Sadia Himu:** writing – original draft, investigation, formal analysis, data curation, methodology. **Md. Faridul Islam:** resources, writing – review and editing. **Shariful Islam:** conceptualization, investigation, funding acquisition, writing – original draft, writing – review and editing, visualization, validation, methodology, software, formal analysis, project administration, supervision, data curation, resources. **Md. Nuruddin Miah:** writing – review and editing, supervision. **Md. Jaynal Abedin:** methodology, formal analysis, data curation, resources. **Amena Kibria:** formal analysis, data curation, resources. **Md. Abdus Satter Miah:** supervision, project administration, writing – review and editing, funding acquisition. **Mohammad Nazrul Islam Bhuiyan:** writing – review and editing.

## Funding

This research received financial support from the Research and Development project “Development of Fiber and Antioxidant Rich Cereal‐Based Functional Food” (R&D no. IFST‐09; letter no. 39.02.0000.011.14.169.2023/877; Date: 17.09.2023), Institute of Food Science and Technology (IFST), BCSIR, Dhaka, Bangladesh.

## Disclosure

*Authors Agreement*: All authors have read and approved the final version of the manuscript. Dr. Abdus Satter Miah had full access to all of the data in this study and takes complete responsibility for the integrity of the data and the accuracy of the data analysis.

*Research Involving Humans and Animals Statement*: The authors confirm that the sensory evaluations were carried out using volunteer panelists who were fully informed about the nature and purpose of the assessment and who willingly provided their informed consent to participate. The authors also state that all food samples and ingredients used in the sensory testing were safe, edible, and suitable for human consumption.

## Conflicts of Interest

The authors declare no conflicts of interest.

## Supporting information


**Table S1:** Formulation pattern of fiber and antioxidant source addition level pasta.

## Data Availability

Data will be made available on request.
