# Supplementary material for: Development and Quality Evaluation of Fiber‐Enriched Pasta Incorporating Green Banana Flour and Whole Wheat Flour: Physicochemical, Antioxidant, and Sensory Properties
Source: Food Sci Nutr. 2026 Aug 27;14(9):e72273. doi: 10.1002/fsn3.72273 (PMC13522392; doi:10.1002/fsn3.72273)
Supplement: Supplementary file 1 [file FSN3-14-e72273-s001.docx]

**Supplementary information**

**Development and Quality Evaluation of Fiber-Enriched Pasta Incorporating Green Banana Flour and Whole Wheat Flour: Physicochemical, Antioxidant, and Sensory Properties**

Shariful Islam ^1a^_,_ Nusrat Sadia Himu^2a^, Md. Faridul Islam **^1,3^**_,_ Amena Kibria **^1^_,_** Mohammad Nazrul Islam Bhuiyan ^4^, Md. Jaynal Abedin ^1^**_,_** Md. Nuruddin Miah^2^, and Md. Abdus Satter Miah **^5*^**

**^1^** Institute of Food Science and Technology (IFST), Bangladesh Council of Scientific and Industrial Research (BCSIR), Dr. Qudrat-I-Khuda Road, Dhanmondi, Dhaka-1205, Bangladesh.

**^2^** Department of Biochemistry and Molecular Biology, Sher e Bangla Agricultural University, Dhaka-1207.

^3^ Department of Food Technology and Nutritional Science, Mawlana Bhashani Science and

Technology University, Santosh, Tangail-1902, Bangladesh.

^4^ BCSIR Chattogram Laboratories, BCSIR, Chattogram 4220, Bangladesh.

**^5^** Biomedical and Toxicological Research Institute (BTRI), Bangladesh Council of Scientific and Industrial Research (BCSIR), Dr. Qudrat-I-Khuda Road, Dhanmondi, Dhaka-1205, Bangladesh.

^a^ These authors contributed equally to this work.

**Corresponding authors details:**

Dr. Md. Abdus Satter Miah, Director

Bangladesh Council of Scientific and Industrial Research (BCSIR), Dr. Qudrat-I-Khuda Road, Dhanmondi, Dhaka-1205, Bangladesh.

E-mail: mabdussatter@yahoo.com

**Pasta Formulation Pattern**

**Table-S1: Formulation pattern of fiber and antioxidant source addition level pasta.**

| **Ingredient** | **WCL10%** | **WCL15%** | **WCL20%** | **BCL10%** | **BCL15%** | **BCL20%** |
| --- | --- | --- | --- | --- | --- | --- |
| Refined flour (%) | 90 | 85 | 80 | 90 | 85 | 80 |
| Whole wheat flour (%) | 10 | 15 | 20 | – | – | – |
| Green banana flour (%) | – | – | – | 10 | 15 | 20 |
| Cauliflower (%)* | 2.0 | 2.0 | 2.0 | 2.0 | 2.0 | 2.0 |
| Onion powder (%)* | 2.0 | 2.0 | 2.0 | 2.0 | 2.0 | 2.0 |
| Garlic powder (%)* | 1.0 | 1.0 | 1.0 | 1.0 | 1.0 | 1.0 |
| Tomato paste (%)* | 20.0 | 20.0 | 20.0 | 20.0 | 20.0 | 20.0 |
| Salt (%)* | 1.0 | 1.0 | 1.0 | 1.0 | 1.0 | 1.0 |
| Chili powder (%)* | 0.5 | 0.5 | 0.5 | 0.5 | 0.5 | 0.5 |
| Egg (%)* | 16.0 | 16.0 | 16.0 | 16.0 | 16.0 | 16.0 |

**Note:** Percentages are expressed on a baker's percentage basis, where the total flour (refined flour + whole wheat flour or green banana flour) equals 100%. All others ingredients were kept constant relative to the total flour weight. WCL10%, WCL15% and WCL20%: 10%, 15% and 20% whole wheat flour incorporated pasta, respectively.; BCL10%, BCL15% and BCL20%: 10%, 15% and 20% green banana flour incorporated pasta, respectively.

**Water Uptake Index**

To calculate water uptake index, method was adapted from Dodi et al. (2022), with slight modifications to suit fiber-enriched pasta. Weigh 10 g of dried pasta and boiled in 300 mL of distilled water for the previously determined optimum cooking time (OCT). After cooking, samples were drained, rinsed with cold water, and blotted to remove surface moisture. Every measurement was carried out two times. The weight of the cooked pasta was recorded, and water uptake index was calculated using the following **equation 1**.

Water uptake (%, dry basis) = (W*_cooked pasta_* – W*_dry pasta_*)/W*_dry pasta_* × 100 ………… (1)

Where: W*_dry pasta_* = Initial dry weight of the pasta sample, and W*_cooked pasta_* = Final weight after cooking and blotting

**Cooking Loss**

Cooking loss was measured to evaluate the amount of solid material leached into the cooking water during the pasta cooking process. With slight change, method was adapted from Axenti et al. (2023). 10 g of dried pasta was cooked in 300 mL of distilled water for the previously determined optimum cooking time (OCT). After cooking, the pasta was drained through a 200 mm sieve, and the cooking water was collected. The water was then evaporated in a drying oven at 105 °C until a constant weight was achieved. All measurements were conducted in duplicate, and results were expressed as mean ± standard deviation. The dry residue was weighed and expressed as a percentage of the initial dry pasta weight using the following **equation 2**.

Cooking Loss (%, dry basis) = (W*_dry pasta_*/W*_residue_*) × 100 ……………………. (2)

Where, W_residue_ = weight of solids recovered from cooking water, and W_dry pasta_ = initial weight of uncooked pasta.

**Texture Analysis**

The texture of cooked pasta was analyzed using a texture analyzer (Model: FRTS-100N-I, Imada, Japan) operated with Force Recorder software. The evaluation focused on key textural attributes, including hardness, stickiness, chewiness, cohesiveness, springiness, gumminess, and adhesiveness, with minor adjustments to the testing parameters. A 20 mm diameter flat probe was used to perform two-cycle compression tests, maintaining pre-test and post-test speeds of 5.0 mm/s and 3.0 mm/s, respectively (Islam et al., 2025). For analysis, cooked pasta samples were placed in aluminum dishes and positioned on the testing platform. Each sample was tested three replicates to ensure reliability.

**Mineral Analysis**

The mineral solution was prepared following the procedure described by Islam et al. (2024). Ash samples were dissolved in 40 mL of 10% hydrochloric acid and heated on a water bath for two hours to remove any silica. Additional deionized water was added to dissolve the remaining soluble salts, and the solution was filtered through Whatman No. 1 filter paper into a 100 mL volumetric flask, with the final volume adjusted using deionized water. Sodium (Na) and potassium (K) content were measured using a flame photometer (Jenway, PEP7, St Neots, Cambridgeshire, UK), while magnesium (Mg), phosphorus (P), calcium (Ca), iron (Fe), copper (Cu), and zinc (Zn) were quantified using an atomic absorption spectrometer (ICS-3000, Thermo, USA). Actual concentrations were determined using standard calibration curves for each mineral.

**Water Soluble Vitamin B Analysis**

The analysis of B vitamins in developed pasta samples was conducted following the method described by Rashid et al. (2024). A 2.0 g portion of homogenized sample was mixed with 25 mL of 0.1 N H₂SO₄ and subjected to hydrolysis at 120 °C for 30 minutes. After cooling, the pH was adjusted to 4.5 using 2.5 M sodium acetate (CH₃COONa), followed by the addition of 0.05 g of diastase enzyme. The mixture was incubated overnight at 37 °C to facilitate enzymatic digestion. The final volume was adjusted to 50.0 mL with deionized water, and the solution was filtered through a 0.45 µm syringe filter. A 10 µL aliquot of the filtrate was injected into an HPLC system (HITACHI 5000, Japan) equipped with a reversed-phase C18 column (250 × 4.6 mm, 5 µm particle size). Chromatographic separation was performed under gradient elution using phosphate buffer (mobile phase A) and acetonitrile (mobile phase B). The mobile phase B was programmed as follows: 1% from 0.0-3.0 min, 50% from 3.1-10.0 min, and held at 50% until 16.0 min. The flow rate was set to 0.8 mL/min, and detection was carried out at 210 nm.

**Antioxidant Activity**

**Total phenolic content (TFC):** Briefly, 0.5 mL of 10,000 ppm of each sample extract was mixed with 8 mL DI water and 0.5 mL reagent. After 5 min of room temperature (RT) incubation, 1.0 mL of 10% sodium carbonate solution was added, mixed well, and incubate the reaction mixture at RT for 30 min. 100 μg/mL standard ascorbic acid working solution was used to develop a calibration curve. The resultant reaction mixture absorbance was measured at 765 nm using a double-beam spectrophotometer. The TPC was calculated as mg ascorbic acid equivalent/100 g of dry weight.

**Total flavonoid content (TFC):** In short, 0.2 mL of 100,000 ppm of each sample extract solution mixed with 4.8 mL of deionized water and 2.5 mL reagent mixture (0.325 g AlCl_3_ and 1.0 g CH_3_COONa in 100 mL DI water) and mixed well. Incubated the reaction mixture for 5 min and the optical density was taken at 430 nm using a spectrophotometer. 100 μg/mL quercetin standard stock solution was used to prepare the calibration curve. The TFC data were presented as mg quercetin equivalent/100 g of dry weight.

**Total antioxidant content (TAC):** The reagent mixture for this assay was prepared by mixing sulfuric acid (0.6 M), sodium phosphate (28.0 mM), and ammonium molybdate (0.8%) following the 4:2:4 ratio. 0.5 mL of 10,000 ppm each sample extract, 0.5 mL of H_2_O and 3 mL of reagents was mixed together. For incubation, the reaction mixtures were then placed in an oven at 95 ◦C for 90 min. When the reaction mixture reached at RT, the absorbance of the solution was recorded at 695 nm using a spectrophotometer. Ascorbic acid stock solution (100 μg/mL) was used a standard to build the calibration curve. The results were expressed as mg ascorbic acid equivalent/100 g of dry weight.
